# Protein Arginine Methyltransferase 1: A Multi-Purpose Player in the Development of Cancer and Metabolic Disease

**DOI:** 10.3390/biom15020185

**Published:** 2025-01-27

**Authors:** Daphne de Korte, Menno Hoekstra

**Affiliations:** Division of Systems Pharmacology and Pharmacy, Leiden Academic Centre for Drug Research, Leiden University, Gorlaeus Laboratories, Einsteinweg 55, 2333 CC Leiden, The Netherlands; d.h.de.korte@umail.leidenuniv.nl

**Keywords:** PRMT1, protein arginine methyltransferase, cancer, metabolism, type 2 diabetes, obesity, liver disease

## Abstract

Protein arginine methyltransferase 1 (PRMT1) is the main PRMT family member involved in the formation of monomethylarginine and asymmetric dimethylarginine on its protein substrates. Many protein substrates of PRMT1 are key mediators of cell proliferation and oncogenesis. As such, the function of PRMT1 has been most prominently investigated in the context of cancer development. However, recent in vitro and in vivo studies have highlighted that PRMT1 may also promote metabolic disorders. With the current review, we aim to present an in-depth overview of how PRMT1 influences epigenetic modulation, transcriptional regulation, DNA damage repair, and signal transduction in cancer. Furthermore, we summarize the current knowledge regarding the role of PRMT1 in metabolic reprogramming, lipid metabolism, and glucose metabolism and describe the association of PRMT1 with numerous metabolic pathologies such as obesity, liver disease, and type 2 diabetes. It has become apparent that inhibiting the function of PRMT1 will likely serve as the most beneficial therapeutic approach, since several PRMT1 inhibitors have already been shown to exert positive effects on both cancer and metabolic disease in preclinical settings. However, pharmacological PRMT1 inhibition has not yet been shown to be therapeutically effective in clinical studies.

## 1. Introduction

Protein arginine methyltransferases (PRMTs) constitute a family of methylases that have been detected across a large number of organisms [1,2]. As their name suggests, their primary function is to regulate arginine methylation modification, which is a post-translational modification that typically transpires in the cytoplasm and nucleus of the cell. PRMTs catalyze the transfer of methyl groups to guanidine nitrogen atoms of the amino acid arginine, or arginine residues, on target proteins [3,4]. The donor of the methyl group is S-adenosylmethionine (AdoMet), which is a direct product of methionine [5]. These post-translational modifications control a wide variety of biological processes, such as transcriptional regulation, DNA repair, RNA splicing, and the immune response.

In mammals, three classes of methylarginines have been discovered: monomethylarginine (MMA), symmetric dimethylarginine (SDMA), and asymmetric dimethylarginine (ADMA). Linking an individual methyl group to a terminal nitrogen atom creates monomethylarginine (MMA) [6]. Connecting one methyl group to the nitrogen atoms of both guanidines at the end of arginine produces SDMA, and ADMA is generated when two methyl groups are coupled to one terminal nitrogen atom.

**Figure 1:**
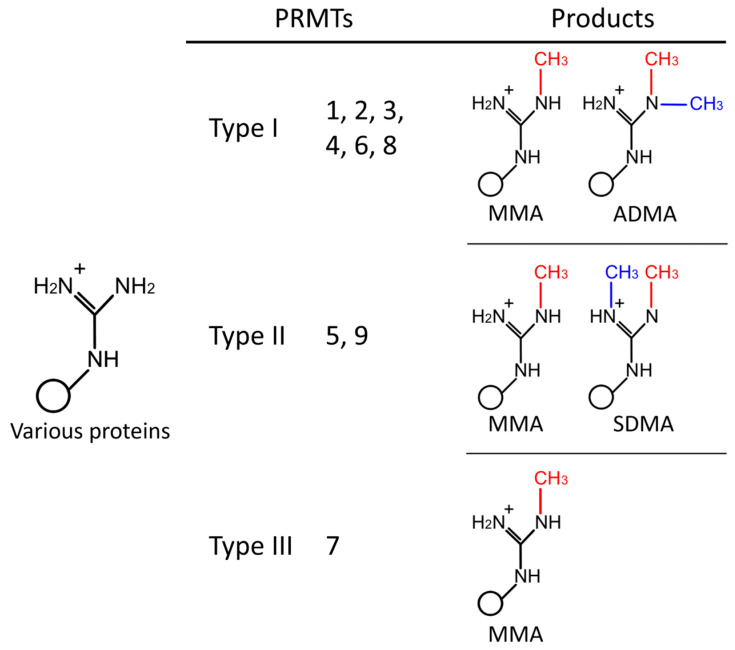
The three types of protein arginine methyltransferases. Each PRMT catalyzes the formation of MMA on one of the terminal guanidine nitrogen atoms of the arginine in target proteins. Type I PRMTs subsequently convert MMA into ADMA, whereas type II PRMTs generate SDMA. Type III PRMTs are only able to produce MMA.

Since the first discovery of PRMTs in the 1990s, extensive research has been performed to map the functions and isoforms of these enzymes. Currently, we know that the PRMT family consists of nine members that can be categorized into three major types: type I, II, and III (Figure 1) [5,7]. Every member of the PRMT family catalyzes the formation of the intermediate product MMA. Consequently, type I PRMTs (PRMT1–4, PRMT6, and PRMT8) generate ADMA and type II PRMTs (PRMT5 and PRMT9) form SDMA. Only type III (PRMT7) solely produces MMA [4,6,8]. ADMA represents the most common form of arginine methylation in cells, followed by SDMA and MMA, respectively [9].

Notably, 85% of the ADMA marks on proteins are generated by PRMT1 [10]. Like all type I PRMT structures, PRMT1 contains two functional domains: the Rossmann fold, otherwise known as the AdoMet binding pocket, at the N-terminus, and the β-barrel domain at the C-terminus [11]. The N-terminal part of the β-barrel domain also constitutes an α-helical dimerization arm that connects with the N-terminus of the AdoMet binding domain. This dimerization arm is critical for PRMT’s ability to dimerize and execute its methyltransferase activity [12]. Even though all PRMT1 proteins conform to this characteristic structure, seven isoforms (PRMT1-v1–PRMT1-v7), yielded by alternative splicing, have been discovered [13]. The differences between isoforms lie in the varying sequences and lengths of the N-terminal domain and influence the specificity of the substrate and activity of the enzyme itself.

Arginine 3 of histone H4 (H4R3) is one of the first PRMT1 substrates discovered [14]. H4R3 methylation is crucial for transcriptional activation and maintenance of active chromatin modifications [15]. Besides methylation of histone H4, PRMT1 is mainly known to methylate arginine residues, specifically “RGG” motifs [16]. Moreover, PRMT1 plays pivotal roles in many other biological processes and cellular pathways. One of the primary functions of PRMT1 is mediating embryogenesis and development. Various research has shown that PRMT1 knockout results in early lethality in mice embryos and developmental defects in zebrafish embryos [17,18]. Furthermore, PRMT1 is active in many cell signaling pathways, including the Wnt pathway, Forkhead box O (FOXO) pathway, and nuclear hormone receptor pathways. All of these pathways control the survival of cells [19,20]. Other proteins that are methylated by PRMT1 are active in DNA damage repair pathways. PRMT1 dysfunction is, therefore, characterized by DNA damage and genetic instability. Many studies have also illustrated that PRMT1 influences transcriptional and translational regulation, RNA splicing, and immune responses [19].

The involvement of PRMT1 in so many processes makes this enzyme a particularly interesting research subject. Scientists have already extensively studied the roles and functions of PRMT1 in cancer. However, more recently, PRMT1 has also become a subject of interest in research related to metabolic processes and pathways. This review aims to provide an overview of our current knowledge regarding the established role of PRMT1 in cancer and to spark interest in the upcoming findings related to PRMT1 from the metabolic field.

## 2. PRMT1 Dysregulation in Cancer

Cancer is one of the most predominant causes of death worldwide, with approximately 10 million deaths per year [21]. Usually, this disease is the consequence of mutations in the genome. The most common forms of cancer are breast, lung, colon, rectum, and prostate cancers. Major risk factors for cancer development include alcohol consumption, physical inactivity, and an unhealthy diet. Avoiding these risk factors can prevent 30–50% of cancers and reduce the burden on healthcare. Besides these risk factors, genetic alterations can also be inherited, or they can occur spontaneously from replication errors or DNA damage due to environmental carcinogens [22]. These inherited or induced DNA alterations can dysregulate the structure and function of genes [23]. Such mutations are often inconsequential, but a neoplasm can arise when it occurs in the coding and noncoding parts of the genome. In general, tumorigenesis is viewed as a multistep process, because it is often not just a single mutation that drives tumor development [24]. However, there are certain similar mutations that occur frequently in many types of cancer. For example, a mutation in tumor suppressor genes can cause an uncontrollable replication of cells, even outside of their ordinary niche. Changes in methylation of histones, transcription factors, and DNA damage repair proteins are a major promoter of oncogenesis. These methylation changes can potentially be instigated by PRMT1 as high expression of PRMT1 has been documented to induce a proliferation and transformation of cancer cells in several types of cancer. Moreover, PRMT1 upregulation has also been shown to serve as an indicator of disease progression and aggressiveness [25,26].

### 2.1. PRMT1-Mediated Epigenetic Modulation Aggravates Cancer Severity

**Figure 2:**
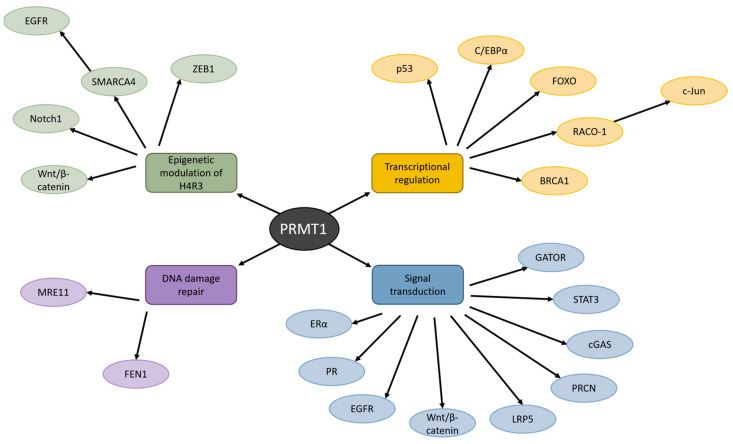
The influence of PRMT1 on various cellular processes in cancer. PRMT1 manages H4R3, transcriptional regulation, DNA damage repair, and signal transduction by methylating a wide variety of proteins. Dysregulation of these processes by PRMT1 has been detected in many types of cancer.

PRMT1 has a wide variety of substrates and is predominantly known for asymmetric dimethylation of histone H4R3 [27]. This catalysis process is essential for the activation of chromatin and transcriptional control [28]. Aberrant epigenetic alterations, such as hypermethylation and anomalous histone modifications, have been detected in promoter regions of tumor-suppressor genes, and evidence suggests that PRMT1 may be a prominent instigator of these alterations and the progression of cancer (Figure 2) [27,29]

This was shown in a study by Zhao and colleagues, who investigated the effect of PRMT1 in esophageal squamous cell carcinoma (ESSC) [29]. Indeed, they reported that PRMT1 methylated histone H4R3 in tumor-initiating cells (TICs), which modified the genome and caused transcriptional activation of downstream genes. Moreover, overexpression of PRMT1 in esophageal TICs augmented their stem cell-like properties, tumorigenicity, and chemoresistance. Furthermore, this study highlighted that knockdown of PRMT1 reduced the ability of ESCC cells to self-renew. RNA-seq data also revealed that genes of the Wnt/β-catenin signaling pathway were upregulated during PRMT1 overexpression in ESSC cell lines. This pathway is characterized by its stem cell and self-renewal properties. During validation of these RNA-seq data, the researchers also discovered that PRMT1 overexpression elevated the expression of Notch1, a protein whose signaling highly contributes to development and homeostasis in various tissues [30]. Divergent Notch1 signaling can lead to cancer and other diseases. However, the Wnt/β-catenin and Notch signaling pathways are not the only ones affected by H4R3 methylation.

A study on colorectal cancer (CRC) conducted by Yao et al. revealed that methylation of H4R3 by PRMT1 recruits SMARCA4, an ATPase subunit of the SWI/SNF chromatin remodeling complex, which enhances EGFR signaling and subsequently induces the ability of CRC cells to proliferate, form colonies, and migrate [27]. In accordance, elevated EGFR expression due to upstream activation by PRMT1 and SMARCA4 leads to a shorter overall survival of CRC patients. Furthermore, inhibition of PRMT1 severely suppresses CRC cell growth. Another characteristic of cancer cells that PRMT1-mediated H4R3 methylation regulates is the epithelial–mesenchymal transition (EMT) and senescence in breast cancer [28]. Methylation of H4R3 by PRMT1 at the ZEB1 promoter induces its transcription. Activation of ZEB1 has been shown to initiate EMT, migration, and invasion in breast cancer cells. ZEB1 also contributes to the suppression of oncogene-induced senescence. Furthermore, abrogation of PRMT1 drastically reduces cell growth by arresting the breast cancer cells in the G2/M phase, which leads to erroneous cell division and growth.

### 2.2. Aberrant Methylation Disrupts Tumor Suppressor and Oncogene Transcription Factors

PRMT1 has also been reported to regulate the activity of transcription factors involved in cancer (Figure 2). Methylation of transcription factors by PRMT1 can significantly alter their stability, resulting in the enhancement or disruption of their transactivation function [20]. This activation or repression of transcriptional function can lead to a dysregulation of the cell cycle and promote cancer development.

One of the most important transcription factors and tumor suppressors is the protein p53 [31]. p53 responds to a variety of stress signals and strictly supervises numerous cellular processes, including senescence, DNA repair, cell cycle arrest, and apoptosis. Mutations in p53 have been detected in about 50% of cancer cases in humans, making it the most frequently mutated gene in cancer [32]. Recently, Liu et al. brought to light that PRMT1 controls the functionality of the transcription factor p53 in breast cancer [33]. Given the high occurrence rate of p53 mutations in cancer, it would not be unlikely that this is also the case in other cancers, although not many studies have covered the interplay between PRMT1 and p53 yet. Liu’s research group found that the transcriptional activity of p53 was suppressed by the direct binding of PRMT1 to p53. This led to a reduction in the activity of proteins downstream of the p53 signaling cascade. Inhibition of PRMT1, which allowed p53 to remain active, attenuated breast cancer cell growth and induced cellular senescence [33].

Another essential transcription factor that contributes to managing the expression of cell-cycle regulators is C/EBPα [34]. C/EBPα is the predominant member of the CCAAT/enhancer binding proteins (C/EBP) complex and functions as a tumor suppressor in malignant tumors by inhibiting cell proliferation [35]. Post-translational modifications, such as methylation, can block the function of C/EBPα. Overexpression of PRMT1 has been detected in human breast cancer patients [34]. Moreover, PRMT1 was identified as a component in the C/EBPα complex, and methylation of C/EBPα inhibited the interaction between C/EBPα and corepressor HDAC3, leading to the activation of cyclin D1 expression and rapid tumor cell growth. Upon complete abrogation of endogenous PRMT1, and with the use of a PRMT1-specific inhibitor, cell viability was attenuated in triple-negative breast cancer cell lines (TNBC1 and TNBC2), one of the most aggressive forms of breast cancer.

The transcription factor FOXO was already an attractive target in cancer research, due to its ability to activate DNA repair, cell cycle arrest, and apoptosis [36]. FOXO dysfunction and dysregulation can lead to genomic instability and tumor formation from failed reparation of damaged DNA and uncontrolled cell division [36]. In response to cellular growth factors and insulin, FOXO is activated by Akt-mediated phosphorylation, and stress sensitivity, proliferation, and cell survival are promoted. In the absence of growth factors and insulin, FOXO is not phosphorylated and activated, cell cycle progression is arrested, and cell death is induced. PRMT1-mediated methylation of FOXO1 blocks phosphorylation by Akt [37]. This would suggest that inactivation of FOXO1 by PRMT1 methylation in cancer cells might attenuate cancer progression. Interestingly, many papers claim that FOXO is a tumor suppressor as FOXO inhibits tumorigenesis and FOXO loss can enhance tumorigenesis. This would mean that perhaps activation of this transcription factor may be more beneficial [37,38,39]. However, a most-enlightening review by Hornsveld and colleagues extensively covers how FOXO can function in both a tumor-supporting and tumor-suppressing way [40]. It all depends on the cellular context, development, and progression of the tumor. Therefore, because PRMT1 regulates FOXO activity, it is crucial to take into account the cancer type and stage when testing anti-cancer treatments by PRMT1 suppression.

The previously mentioned transcription factors are all classified as tumor suppressors—albeit FOXO dubiously so. However, PRMT1 methylation does not solely impact tumor suppressing transcription factors, but also oncogenic transcription factors like c-Jun [41]. c-Jun is the key enzyme in the AP-1 transcription factor complex and plays an important role in proliferation and oncogenic transformation [42]. Under normal circumstances, c-Jun is activated when RING domain-containing protein RACO-1 dimerizes and links growth factor signaling to AP-1 transactivation. PRMT1 is the enzyme that causes the dimerization of RACO-1 and facilitates interaction with c-Jun. When PRMT1 is abrogated, disabling it from methylating RACO-1, c-Jun target gene expression and AP-1 activity decrease. This identifies PRMT1 as an important regulator of the oncogenic c-Jun/AP-1 complex. Our knowledge of the effect of PRMT1 inhibition on c-Jun function is lacking. However, since c-Jun has also been found to induce metastasis, a major problem for cancer prognosis, it would be interesting to study the relationship between these proteins and tumor cell migration [43].

One substrate protein of PRMT1 that is defined as a transcription factor but also functions in DNA damage repair is BRCA1 [44]. The transcription factor BRCA1 activates the DNA damage response, and a mutation of its gene has been detected in approximately half of hereditary breast cancer cases. In addition, it is believed that resistance of cancer cells against irradiation therapy arises from the cells’ ability to instigate DNA repair mechanisms [45]. Montenegro’s research group revealed that ionizing radiation (IR) causes methylation of BRCA1 by PRMT1, and that PRMT1 controls the cellular localization of BRCA1, which is essential for its DNA damage repair function. Moreover, BRCA1 methylation promoted DNA homologous recombination repair activity and improved the breast cancer cells’ defense against IR. It would be interesting to additionally investigate the relationship between PRMT1 and BRCA2, but all current evidence already suggests that PRMT1 inhibition may be an appealing therapeutic approach in cancer.

### 2.3. PRMT1 Aids Chemoresistance by Inducing DNA Damage Repair Pathways

A major cause of cancer is DNA damage as a result of environmental sources [46]. These damage sources can be physical (ionizing radiation, ultraviolet light), chemical (industrial chemicals, chemotherapeutic drugs), or biological (viruses, bacteria) and introduce single- or double-stranded DNA breaks, or DNA crosslinking. When the DNA is not repaired faultlessly, it can cause mutations in oncogenes and tumor suppressor genes. Chromosomal aberration of these genes can lead to the development of cancer. Therefore, the correct functioning of the DNA damage repair mechanism is crucial for the maintenance of genome integrity and healthy cells. PRMT1 has been found to regulate DNA damage repair pathways and dysregulate these in cancer environments (Figure 2).

A transcriptome analysis after pharmacological PRMT1 inhibition for one, two, and three days, performed by Giuliani et al. in PATC53 and PANC1 cells in vitro, illustrated that PRMT1 mediates a variety of DNA damage response pathways [47]. They observed a severe disruption in gene expression and a time-dependent increase in the number of affected genes. The genes associated with pathways involved in DNA repair were most-prominently downregulated after two and three days of inhibition. Specifically, the genes of the entire family of the Fanconi anemia DNA repair pathway were downregulated upon PRMT1 suppression. The rare genetic disorder Faconi anemia is caused by a defect in the homologous recombination DNA repair pathway, but also various proteins with the function of repairing damaged DNA upon exposure to alkylating agents, cytotoxic drugs, and irradiation [48]. This disorder is associated with a predisposition for tumors [49].

One complex that has been studied in multiple cancers is the MRE11-RAD50-NBS1 (MRN) complex [50]. MRN functions in repairing double-stranded DNA breaks to conserve genome stability, with MRE11 as its key component [51]. MRE11 is the only protein of the MRN complex that PRMT1 interacts with, likely due to its glycine-arginine-rich (GAR) motif. Additionally, increased expression of MRE11 in certain cancer types has been illustrated in patients undergoing irradiation therapy [52]. These elevated MRE11 levels were associated with treatment resistance and poor survival. A study by Boisvert et al. demonstrated that suppression of PRMT1 decreased the function of MRE11, resulting in DNA damage [53].

Another DNA repair protein that PRMT1 targets is Flap endonuclease 1 (FEN1) [54]. FEN1 is a key enzyme in the Base Excision Repair pathway and plays a central role in cellular DNA metabolism by regulating Okazaki fragment processing, DNA replication, double-stranded DNA break repair, and long-patch base excision repair to maintain genome stability [55,56]. Upregulation of PRMT1 can lead to an abundance of post-translational modifications on, and overexpression of, FEN1 [54]. FEN1 has been found to be pivotal for drug resistance and proliferation in several types of cancer, because it repairs damaged DNA after chemotherapy. Knockdown of PRMT1, which destabilizes FEN1, can induce DNA damage and apoptosis of cancer cells. Altogether, this makes PRMT1 inhibition an appealing treatment option to attenuate cancer cell proliferation and survival by disrupting DNA damage repair proteins. However, the impact of PRMT1 inhibition on DNA damage repair in healthy cells should be investigated during our search for a suitable anti-cancer treatment, as this may affect what type of therapy will be used. In other words, should a PRMT1 inhibitor target the tumor, or can it be administered systematically?

### 2.4. PRMT1 Activity Interferes with Signal Transduction Pathways in Cancer

Proper management of signal transduction and molecular networks is pivotal for cell growth and survival. These molecular signaling pathways are often disrupted in cancer cells, allowing them to proliferate rapidly and uncontrollably [57]. An abundance of research has reported that many different signaling pathways in cancer cells are dysregulated due to PRMT1-mediated methylation (Figure 2).

Nuclear receptors, such as the estrogen receptor (ER), progesterone receptor (PR), and epidermal growth factor receptor (EGFR), mark the starting line of signaling cascades and are often dysregulated in tumors. Specifically in breast cancer, divergent ER and PR expression can participate in several aspects of tumorigenesis. Estrogen transduction, led by the ERα, promotes gene transcription and cell growth in breast epithelial cells and various other tissues by inducing growth factor-dependent kinases and adaptor proteins. Consequently, this causes the activation of numerous downstream signaling proteins, including Akt, MAPK, p21ras, and protein kinase C [58]. Abnormal responses to estrogen signaling are particularly prominent in breast cancer. This deviant estrogen signal transduction seen in breast cancer can be the cause of ERα hypermethylation by PRMT1, resulting in cytoplasmic localization of the ERα and subsequent cancer progression. Indeed, PRMT1 inhibition can undermine ERα signaling [59].

Progesterone is also an important hormone in the context of breast cancer. The activity of its receptor is tightly controlled by several post-translational modifications that influence subcellular localization, sensitivity to hormones, protein stability, and cofactor interactions [60]. PRMT1 functions as a cofactor in regulating the progesterone pathway. Methylation of PR by PRMT1 leads to a decrease in the stability of this receptor, which enhances its recycling, followed by its transcriptional activity. Suppression of PRMT1 using an siRNA can attenuate proliferation and migration of breast cancer cells and is predicted to cause longer survival of breast cancer patients.

Overexpression of PRMT1 has been detected in all breast cancer subtypes in comparison to healthy breast tissue, although it does not always affect the estrogen or progesterone hormone signaling cascades. For example, in TNBC where neither of these hormone receptors are expressed, PRMT1 mediates EGFR and Wnt signal transduction [61,62]. Researchers showed that PRMT1 depletion has a direct effect on *EGFR* mRNA by decreasing its expression, which consequently reduces protein expression of EGFR [62]. These results reveal that PRMT1 not only enhances EGFR signaling by methylating H4R3 but also regulates EGFR expression at the promoter region [27]. Furthermore, in the Wnt signaling pathway, *LRP5* and *PRCN* (Porcupine) genes and LRP5 protein are expressed less in response to PRMT1 suppression [62]. Porcupine protein expression was not analyzed. The same research group also confirmed that PRMT1 is a promoter of the Wnt pathway by investigating the expression of *AXIN2*, *APCDD1*, and *NKD1*, three target genes of Wnt. Inhibition of PRMT1 decreased Wnt activity in a dose-dependent manner. These findings, thus, highlight that PRMT1 controls the Wnt pathway by also activating the promoter of various genes in this cascade, whereas Yao et al. had discovered SMARCA4 as a positive regulator of Wnt [27]. Besides its role in breast cancer, overexpression of PRMT1 also promotes the Wnt/β-catenin pathway in other types of cancer, such as gastric cancer [63]. β-catenin functions as a downstream signal transducer in the Wnt/β-catenin pathway and can induce cancer cell migration and metastasis [63,64]. Wang and colleagues reported that PRMT1 abrogation and inhibition caused a suppression in gastric cancer cell proliferation, tumorigenesis, migration, and invasion [63]. PRMT1 exerted this effect by binding and recruiting MLX-interacting protein (MLXIP) and Kinectin 1 (KTN1) to the β-catenin promoter.

PRMT1 expression also plays a substantial role in tumor immunity. The microenvironment of the tumor can become more immunosuppressive when the expression of PRMT1 is elevated [65]. In addition, in various types of cancer, PRMT1 presence is positively correlated with neoantigens, microsatellite instability, and mutational burden of the tumor. One of the pathways that is critical for tumor immunity is the cGAS/STING innate immunity pathway [66]. Tumors have the ability to escape immune surveillance by impairing cGAS transduction in cancer cells to accelerate tumorigenesis [67]. PRMT1 has been found to methylate cGAS and prevent it from dimerizing, which attenuates the cGAS/STING signaling cascade in cancer cells [66]. PRMT1 inhibition and deficiency can reverse this effect and induce the cGAS/STING pathway to promote the gene transcription of type I and II interferon response factors. Subsequently, the number of tumor-infiltrating lymphocytes is increased and expression of tumoral PD-L1 is augmented. The favorable effects of PRMT1 inhibition on cGAS/STING pathway activation were particularly prominent following radiation therapy. PRMT1 is also involved in tumor immunity by decreasing the activity and number of CD8^+^ T cells [68]. Both genetic and pharmaceutical abrogation of PRMT1 has been shown to promote interferon responses of cancer cells due to lack of H4R3 histone modification. With an additional PD-1 blockade, the proportion of intratumoral CD8^+^ T cells was elevated in conjunction with IFNγ^+^CD8^+^ T cells.

Numerous studies have illustrated that the JAK/STAT3 pathway is constitutively active in hepatocellular carcinoma (HCC) and is involved in the development and progression of this disease. Zhang et al. revealed that overexpression of PRMT1 caused an increase in STAT3 signaling [69]. PRMT1 expression and activation of the STAT3 signaling pathway was associated with poor prognosis and cancer recurrence in addition to tumor cell proliferation, differentiation, size, and migration, highlighting the essential role PRMT1 plays in tumorigenesis.

One more interesting pathway in cancer that can be disrupted by PRMT1 methylation is the pathway of the mechanistic target of rapamycin complex 1 (mTORC1). mTORC1 senses various signals, such as amino acids, from the cell’s environment and exerts a key function in cell growth and metabolism [70]. One protein complex that is crucial for linking these signals to mTORC1 is the GATOR2 complex, which is mediated by PRMT1 methylation [71]. PRMT1 methylates the WDR24 component of GATOR2 upon phosphorylation by cyclin-dependent kinase 5 (CDK5). Overexpression of PRMT1 has been shown to amplify mTORC1 signaling in patients with HCC. Moreover, HCC cell proliferation and tumor growth thrive on a highly active CDK5-PRMT1-WDR24 pathway. Thus, disrupting this pathway by PRMT1 inhibition may lead to HCC cell cycle arrest and a decrease in tumor size.

## 3. The Role of PRMT1 in Metabolic Disorders

Obesity has been a major health challenge worldwide for many years now and will only continue to become a pandemic-level global health threat over the years to come [72,73]. Excess body weight significantly increases the risk of many metabolic diseases, including fatty liver disease, dementia, cardiovascular disease, and type 2 diabetes [74]. Cardiovascular disease is already the leading cause of death worldwide, with an estimated 17.9 million deaths per year [75]. Diabetes is a chronic disease resulting from the inability of the β-cells of the pancreas to produce a sufficient amount of insulin or failure of the body to effectively utilize the insulin it creates [76]. This defect in the regulation of insulin and glucose levels can lead to severe degeneration of a variety of organs in the body, including blood vessels and nerves. Organ damage and elevated blood glucose levels can also cause kidney diseases and cardiovascular diseases, which are a major cause of death worldwide. In 2021, it was estimated that diabetes-related healthcare cost 966 billion U.S. dollars, globally [77]. These expenses will only increase in the upcoming years due to the growing population and people adopting unhealthier lifestyles. By 2045, global healthcare spending is expected to increase to one trillion U.S. dollars. Currently, diabetes therapy is focused on pharmacological interventions such as gluconeogenesis inhibitors, insulin sensitizers, and weight-loss medications [78,79]. However, these treatments cannot control the disease completely, and a novel, multifaceted therapy may, therefore, be required. It is, thus, of utmost importance to put more effort into the research of diabetes and other metabolic-related diseases to alleviate this heavy burden on our economy, society, and healthcare. For a broader context regarding PRMTs in cardiovascular disease, readers are referred to the recent review by Zhang et al. [80]. Although their review encompasses similar topics, our work focuses specifically on PRMT1, providing a more detailed examination of its role in metabolic reprogramming, lipid metabolism, and glucose metabolism.

### 3.1. Metabolic Reprogramming Induced by PRMT1 Causes Cancer and Metabolic Diseases

In recent years, scientists have begun to illustrate the role of PRMT1 in metabolism. Even in cancer, this enzyme functions in metabolic reprogramming, which can influence the immune microenvironment. For example, PRMT1-mediated metabolic reprogramming is required for leukemia progression [81]. PRMT1 expression is upregulated in acute megakaryocytic leukemia to enhance the usage of glucose for extra nutrients in the cancerous cells, and PRMT1 expression reduces fatty acid oxidation. Consequently, the suppression of fatty acid oxidation reduces propionylated histones, meaning that metabolic reprogramming by PRMT1 also causes epigenetic changes during leukemia progression.

**Figure 3:**
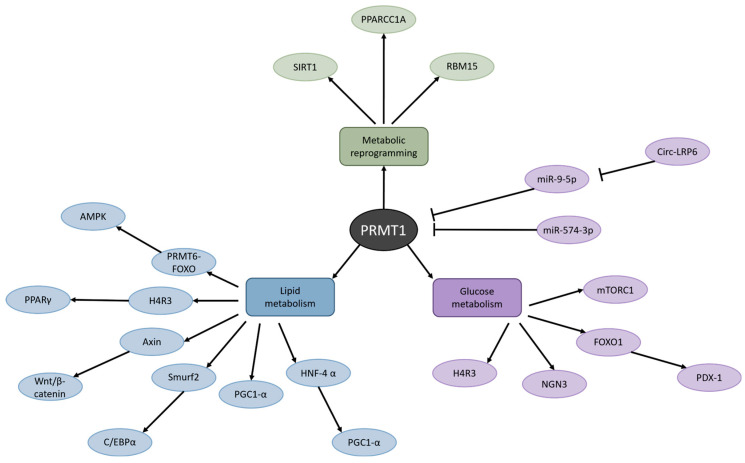
Metabolic routes affected by PRMT1. PRMT1 dysfunction has been detected in metabolic reprogramming, lipid metabolism, and glucose metabolism, which is associated with numerous pathologies such as obesity, liver disease, and type 2 diabetes.

Another study that highlighted the effect of PRMT1 on fatty acid metabolism in the context of cancer was performed recently by Yan and colleagues in HCC [82]. They detected that, in HCC cells with PRMT1 knockdown, cell viability, migration, and invasion were decreased. In addition, their results illustrated an enrichment of PRMT1 in amino acid and drug metabolism and fatty acid degradation. However, that is not the only discovery they made. Fascinatingly, a DisGeNET database analysis revealed that various metabolic diseases, such as fatty liver disease, drug-induced liver disease, and liver injury, were associated with PRMT1 as well [82]. This suggests that PRMT1 signaling likely is involved in many different types of diseases and could potentially be an interesting target in metabolic diseases (Figure 3). Since dysfunctions in metabolism are having a great impact on global health, science and society would benefit immensely if researchers interested in PRMT1 widened their horizons and shifted their views from cancer to the metabolic field.

### 3.2. PRMT1 Is a KEY Regulator of Lipid Metabolism

As can be appreciated from Figure 3, PRMT1 also plays an important role in lipid metabolism. A study conducted by Choi et al. in 2021 elucidated the mechanism of PRMT1 in adipose tissue under a metabolic-disease state [83]. The researchers detected a profusion of PRMT1 expression in white adipose tissue (WAT) in mice fed a high-fat diet that mimics the diet of humans with obesity. Knockdown of the *Prmt1* gene in adipocytes led to a reduction in fat mass while the overall body weight of the mice remained constant. Moreover, the AMPK pathway was induced via the PRMT6-FOXO pathway upon *Prmt1* depletion in WAT, resulting in decreased lipid droplet size, and a promotion in mitochondrial lipid catabolism and lipophagy. AMPK is a master regulator of cellular and organismal metabolism and energy homeostasis [84]. Activation of AMPK positively regulates, for example, fatty acid oxidation, and negatively regulates lipid synthesis and gluconeogenesis. AMPK was already considered a potential therapeutic target against disorders such as type 2 diabetes and obesity. Now that it has been revealed that PRMT1 may control AMPK as well, it makes PRMT1 all the more interesting to study in metabolic diseases. Interestingly, Choi et al. also noted that the *Prmt1* FKO mice in their research displayed insulin resistance and impaired insulin signaling [83]. They speculate that this resistance was caused by the suppression of autophagy in adipocytes. The adipocyte-specific *Atg3* KO mice in a study by Cai et al. also showed resistance to insulin [85]. In contrast, adipocyte-specific *Atg7* KO mice used in studies by other researchers exhibited increased insulin sensitivity [86,87]. It could be argued that the *Atg7* KO mouse model is flawed, because the aP2-Cre system used to generate this model causes ubiquitous aP2 expression, which could alter the metabolic phenotype [83]. Given these opposing findings, it is important to conduct further research into the impact of PRMT1 inhibition on insulin signaling and resistance. Choi and colleagues studied the effect of complete *Prmt1* depletion in WAT alone. However, if an anti-PRMT1 drug was to be used in humans, it would likely be in the form of an inhibitor, meaning that PRMT1 would be suppressed in all tissues, and the inhibition would probably not be 100%. Thus, it is necessary to use another approach to investigate the effects of PRMT1 inhibition in disease, something more relevant to the human patient we are aspiring to treat. A first and foremost strategy could be to administer PRMT1 inhibitors to diabetic test animals. An extensive, if not dated, review about the use of animal models in diabetes research suggests that type 2 diabetes in cats closely resembles numerous characteristics of this condition in humans [88]. In addition, it is essential to study the long-term impact of PRMT1 inhibition. Lastly, Choi et al. mention that they found a reduction in adipocyte size upon *Prmt1* depletion, although they do not examine the effect of PRMT1 on adipogenesis, the process by which adipocytes develop as adipose tissue [83,89].

Zhu et al. have published a paper where they investigated how PRMT1 influences adipogenesis [90]. With both a gain- and loss-of-function model, they highlighted that, indeed, PRMT1 is vital for adipogenesis. Methylation of H4R3 by PRMT1 activates the transcription factor peroxisome proliferator-activated receptor-γ (PPARγ), a known mediator of adipogenesis. Of note, PPARγ activation is also responsible for insulin sensitization and the promotion of glucose metabolism [91]. Furthermore, PRMT1 promotes Axin levels, which attenuates Wnt/β-catenin signaling and induces adipogenic differentiation [90]. The Wnt/β-catenin signaling pathway has been detected to suppress PPARγ and C/EBPα expression, leading to the inhibition of adipogenesis. In addition, PRMT1 can decrease Smad ubiquitination regulatory factor 2 (Smurf2) levels, consequently stabilizing C/EBPβ and promoting adipogenesis [90]. Thus, PRMT1 controls adipogenesis via different pathways, and since there is still so little knowledge about PRMT1 in the metabolic field, it cannot be excluded that PRMT1 regulates adipogenesis via other pathways as well.

Research has also revealed that there is a distinction between PRMT1 isoform functions in adipocyte browning and thermogenesis [73]. Both PRMT1v1 and PRMT1v2, but especially PRMT1v2, participate in the induction of thermogenic fat. Activating the adipocytes in this thermogenic tissue has been shown to be a promising treatment against obesity. PRMT1v2 induces these cells by interacting with PGC1-α, a key transcriptional co-activator that regulates mitochondrial biogenesis. This knowledge can be used in future research to aid in the discovery of a new treatment for metabolic disorders that lead to obesity.

Another pathology that is mediated by PRMT1 is non-alcoholic fatty liver disease (NAFLD) [92]. Abundant intake of triglycerides via the diet causes PRMT1 overexpression. Interestingly, obese patients often exhibit protein hypomethylation in their livers [93]. Recently, a study on the impact of PRMT1 on hepatic steatosis revealed that induction of PRMT1 relieved diet-induced hepatic steatosis in both obese mouse models and obese human patients [92]. PRMT1 exerts this function by recruiting HNF-4α to the promoter of PGC-1α, which induces PGC-1α-mediated fatty acid oxidation. These results argue that a potential treatment strategy against NAFLD would be to activate PRMT1 or PRMT1/HNF-4α/PGC-1α signaling.

This is not the only research that would suggest activation of PRMT1 as therapeutic strategy. Also in alcohol-induced liver injury, Zhao et al. discovered that PRMT1 reduced apoptosis caused by oxidative stress in hepatocytes, promoting cell survival [94]. Knockout of PRMT1 in alcohol-fed mice led to increased fibrosis, inflammation, and cell death in the liver. PRMT1 was also dephosphorylated at Serine 297 due to alcohol consumption. Serine 297 is essential for the ability of PRMT1 to perform its methylation function. This evidence would also imply that PRMT1 induction could be a potential alleviator of a metabolism-related disorder in the liver. Therefore, it is quite fascinating that another study on liver cirrhosis suggests otherwise. Liver cirrhosis is the end stage of steatosis, which can be caused by both an alcoholic and non-alcoholic diet [95]. Yan and colleagues reported that expression of PRMT1 was elevated in hepatic stellate cells (HSC) and hepatocytes of fibrotic livers [96]. Fibrotic genes were induced when PRMT1 was overexpressed in test conditions, and PRMT1 suppression by both genetic knockdown and pharmacological intervention reversed this effect. In addition, pro-fibrotic mediators, such as TFG-β, and pro-inflammatory NF-κB signals were reduced in an HSC-specific *Prmt1* knockout, according to RNA-seq analysis. Interestingly, inhibition with the type 1 PRMT inhibitor PT1001B resulted in reduced PRMT1 activity and alleviated liver fibrosis [96]. Indeed, the hepatic inflammatory response was suppressed upon inhibition in the fibrotic model. Contrastingly, hepatocyte-specific *Prmt1* knockout showed no effect on liver fibrosis at all. This again highlights the importance of a suitable model to analyze PRMT1 function and inhibition in metabolic diseases. Moreover, this indicates that the stage of the disease is an important consideration when developing an appropriate therapy.

### 3.3. PRMT1 Dysfunction Disrupts Glucose Metabolism

Besides playing a role in lipid metabolism, PRMT1 has also been found to be dysregulated in glucose metabolism (Figure 3). In this case, dysfunction of PRMT1 is most prominently translated into diabetes—specifically, type 2 diabetes. One of the primary instigators of diabetes is loss of functional β-cell mass [97]. β-cell failure can even predict the onset and progression of diabetes. Moreover, a reduction in β-cell mass due to dedifferentiation has been found to degrade systemic glucose homeostasis [98]. This knowledge motivated Kim et al. to investigate how β-cells conserve their mature identity [99]. Their research revealed that PRMT1 plays a major role in the pathogenesis of diabetes. Knockout of the *Prmt1* gene in β-cells induced diabetes in both fetal and adult mice. Moreover, after *Prmt1* was deleted in the β-cells of adult mice, all methylation of H4R3 and β-cell identity were lost instantly. This suggests that regulation of chromatin accessibility is crucial for the maintenance of β-cell identity and that PRMT1 is the main mediator of this process. Lee and colleagues also illustrated the importance of PRMT1 in the development of the pancreas [100]. The pancreas of pancreatic progenitor cell-specific *Prmt1* knockout (PKO) mice was reduced in size, which was paralleled by a decreased β-cell mass. The growth of the PKO mice was attenuated, and these mice exhibited an extremely diabetic phenotype. A mechanistic study showed that PRMT1 is also required for the regulation and destabilization of NGN3 [100]. During the development of the pancreas in mice, it is necessary that NGN3 is expressed for 24 h, after which it must degrade rapidly again. In the PKO mice, NGN3 expression was sustained for a longer period of time, causing defects in endocrine and exocrine cell development. Together, these studies highlight the role of PRMT1 in the development and maintenance of the pancreas and specifically β-cells, which are necessary for the production of insulin and glucose metabolism. Understanding the influence of PRMT1 on the first stages of development may be the key to elucidating the finer details of the pathogenesis of diabetes.

PRMT1 is not only required for the overall maintenance of β-cells in the pancreas but is also involved in the control of specific β-cell functions. More specifically, protein methylation by PRMT1 has been suggested to act as a mediator of insulin secretion [101]. When PRMT1 expression is elevated, insulin secretion and content are low. The mechanism behind this appears to be that PRMT1 controls the intracellular translocation of FOXO1 and PDX-1 and reduces FOXO1 methylation. In other words, high PRMT1 expression decreases glucose-stimulated insulin secretion, resulting in reduced intracellular insulin levels. Moreover, FOXO1 overexpression, caused by PRMT1 methylation, impairs the function of β-cells.

This evidence would imply that PRMT1 is the master regulator of numerous cellular pathways and signaling cascades. However, in a follow-up study, the same research group elucidated the underlying mechanism of PRMT1-mediated FOXO1 regulation [102]. In the time between this group’s findings, it had come to light that microRNA (miRNA) editing may orchestrate the onset and advancement of diabetes [103,104,105]. These miRNAs have the ability to modulate and interfere with the expression of their target genes, resulting in the regulation of cellular functions. At least 14 miRNAs have been detected in the pathogenesis of diabetic nephropathy, and miR-574-3p has been highlighted as a suitable biomarker for type 2 diabetes [101,106,107]. Indeed, evidence illustrated that miR-574-3p was an upstream mediator of PRMT1, and PRMT1 expression in pancreatic β-cells is inhibited by this miRNA [102]. Furthermore, PRMT1 silencing by miR-574-3p alleviated glucose toxicity in β-cell dysfunction, which consequently promoted insulin secretion and cell survival. However, more research needs to be conducted on the effect of miR-574-3p on type 2 diabetes, since no study in animals or humans has been performed yet.

Not only the miRNA miR-574-3p showed an interesting interaction with PRMT1 in the context of diabetes. The ENCORI tool calculated miR-9-5p to have a binding relationship with PRMT1 as well. This prediction was subsequently validated by a luciferase assay [108]. Binding of miR-9-5p to PRMT1 was shown to attenuate PRMT1 activation by Ma et al. However, there is a circular RNA, named circLRP6, also present in the body that has the ability to adsorb miR-9-5p [108]. When circLRP6 binds to miR-9-5p, the miRNA cannot interact with PRMT1 anymore. Thus, PRMT1 is upregulated by adsorption of miR-9-5p by circLRP6. This study also revealed that knocking down circLRP6 ameliorates insulin secretion, because without this circular RNA, miR-9-5p can inhibit PRMT1, which we know is an effective way to improve the secretion of insulin. Taken together, all this evidence suggests that PRMT1 is an interesting target in diabetes treatment. However, it is important to keep in mind that there are other factors in the cell that control PRMT1 function.

Another way in which PRMT1 can impact insulin secretion is through methionine sensing by mTORC1 signaling. mTORC1 responds to, among others, nutrients and growth factors and translates these into cell growth and proliferation [109]. In humans, dysregulation of this protein complex is known to cause a variety of diseases, such as cancer and diabetes [110]. However, inhibiting mTORC1 with rapamycin would not be a sufficient diabetes treatment, since extended rapamycin therapy can lead to insulin resistance [111]. Therefore, inhibiting PRMT1, which influences the mTORC1 signaling pathway, may be a more advantageous strategy to augment insulin sensitivity, although the long-term impacts of PRMT1 inhibition have not been investigated to this day. It is crucial to study these effects before any conclusions about PRMT1 targeting treatment can truly be drawn.

After looking at the effect PRMT1 has on the sensitivity and secretion of insulin, it is important not to forget the counterpart of insulin: glucose. One of the first studies performed in 2007 by Iwasaki and Yada that covered the impact of PRMT1 on metabolism demonstrated that arginine methylation by PRMT1 is necessary for the maintenance of insulin signaling, which is followed by the transport and uptake of glucose in skeletal muscles [112]. Although this research implied that insulin translocates PRMT1 while no other paper has mentioned this characteristic of insulin, the fact that PRMT1 influences glucose metabolism remains true. In 2009, Iwasaki performed another experiment that illustrated that diminished PRMT1 functioning disturbed hepatic insulin signaling and led to excessive gluconeogenesis in the liver [113]. Furthermore, glucose-stimulated insulin secretion was attenuated by PRMT1 impairment. Interestingly, the study by Iwasaki et al. is the only one that has suggested that impairment of PRMT1 attenuated glucose-stimulated insulin secretion. All other aforementioned studies on this topic concluded that PRMT1 suppression activated insulin secretion.

Choi’s research group also disputes Iwasaki’s findings with a PRMT1 knockdown analysis. Their results depicted that hepatic gluconeogenesis and blood glucose levels were reduced when PRMT1 is absent, whereas Iwasaki claimed that reduced PRMT1 functioning causes increased gluconeogenesis [114]. In addition, Choi et al. depicted that the hyperglycemic phenotype of their test animals was remedied upon PRMT1 knockdown. They also revealed the mechanism behind this effect: methylation of FOXO1 by PRMT1 was essential for hepatic glucose metabolism. When FOXO1 is methylated by PRMT1, it becomes activated and induces hepatic gluconeogenesis, leading to an increase in blood glucose levels. Thus, upon PRMT1 inhibition, FOXO1 remains dormant as well, and blood glucose levels stay low. This again highlights that the interaction between FOXO1 and PRMT1 is important for both gluconeogenesis, the aforementioned insulin secretion stimulated by glucose, and β-cell function.

Regulation of hepatic gluconeogenesis is pivotal for the survival of organisms for the maintenance of systemic homeostasis under metabolic stress, and whenever they undergo an extended period of fasting [115]. Therefore, it may be important to not inhibit PRMT1- and FOXO1-mediated gluconeogenesis entirely. A recent study showed that some isoforms of PRMT1 control gluconeogenesis to a varying degree [116]. Specifically, PRMT1v2 expression levels in liver cells and tissue were demonstrated to be higher in comparison with PRMT1v1. Hepatic gluconeogenesis was prominently induced in a gain-of-function PRMT1v2 model. Moreover, PRMT1v2 in particular was induced significantly in hepatic cells of type 1 and type 2 diabetic mouse models. With the implication that different isoforms may influence hepatic gluconeogenesis to a varying degree, it would be interesting to investigate these variants more in-depth to be able to find the most relevant target in diabetes treatment. This research also validated the findings of Choi and colleagues that a deficiency in PRMT1 alleviated diabetic hyperglycemia, providing further evidence that PRMT1 is a promising research topic in the diabetes research field.

## 4. PRMT1 Inhibitors: Promising Therapeutic Agents

Both cancer and metabolism research suggest that inhibiting PRMT1 would probably be the most beneficial therapy option. As such, numerous PRMT1 inhibitors have been developed that display promising anti-cancer and anti-diabetes properties in cell lines and animal models (Table 1). The first general PRMT inhibitor that was described in the literature is AMI-1, a symmetrical sulfonated urea salt, which was discovered in 2004 [117]. It took another decade before the inhibitors furamidine, DCLX069, DCLX078, MS023, PT1001B, and GSK3368715 were developed [118,119,120,121,122]. Of note, not all these inhibitors are PRMT1-specific but instead may target multiple type I PRMTs. All of these inhibitors have been studied in the context of cancer and showed promising anti-cancer results. Later, decamidine was depicted to possess a 2-fold increase in PRMT1 inhibition in comparison to furamidine [123]. GSK3368715 has been tested on patients with solid tumors and diffuse large B cell lymphoma in a phase I trial from October 2018 until March 2021 [124,125]. Unfortunately, this trial was terminated due to lack of clinical efficacy, i.e., variable and limited target engagement at a lower dosage (100 mg) and risk of thromboembolic events at a higher dosage (200 mg). In 2020, the PRMT1-specific inhibitor TC-E-5003 was revealed to be an appealing anti-tumor drug preclinically, especially in combination with the chemotherapeutic drug-loaded injectable NBCA ethyl oleate implant [126]. The inhibitor C7280948, also specific for PRMT1, has been shown to be effective against proliferation, migration, and invasion of colorectal cancer cells [127]. The unnamed compound 9a, a tetrazole derivative synthesized by Sun et al., has not been used in cancer research yet. However, it was demonstrated to downregulate the Wnt/β-catenin signaling pathway after PRMT1 inhibition [128]. Another promising PRMT1 inhibitor may be WCJ-394, which attenuates the TGF-β signaling pathway, resulting in mesenchymal marker downregulation [129]. Lastly, a peptoid-based inhibitor of PRMT1 was developed in 2022, currently named P2 [130]. This inhibitor is able to promote both apoptosis and autophagy, which could make this cytostatic agent less toxic.

## 5. Perspectives and Conclusions

While PRMT1 has demonstrated to be a promising target in various pathologies, ranging from cancer to metabolic diseases, in preclinical settings, limited clinical data are available regarding the therapeutic effect of anti-PRMT1 drugs. Only one of the aforementioned type I PRMT inhibitors, GSK3368715, has been evaluated in a clinical phase I trial, that was eventually terminated due to the occurrence of serious adverse events. This phenomenon may be related to the fact that PRMTs are ubiquitously expressed inside the body. Administering an inhibitor that distributes across all tissues, including healthy ones, is likely to result in off-target effects. Therefore, it would be desirable to develop a tissue-specific inhibitor that only distributes to the diseased cells or organ structures. This can—for instance—be achieved by generating immunoconjugates, which have already been studied in anti-cancer research [132]. In this approach, a selective PRMT1 inhibitor would be coupled to an antibody that is directed against a cancer tissue-specific antigen, facilitating tumor-specific delivery. In metabolic diseases, such as NAFLD and liver cirrhosis, hepatocytes are the most likely target cell type for therapeutic intervention. *N*-acetylgalactosamine (GalNAc) conjugates are currently one of the most-used leading delivery systems applied for hepatic targeting in the clinic, i.e., in siRNA-driven therapies, as the GalNAc moiety facilitates binding to the asialoglycoprotein receptor that is exclusively expressed by hepatocytes [133,134]. This delivery method has originally been introduced on the market for acute hepatic porphyria treatment and may potentially also serve as beneficial approach to selectively deliver PRMT1 inhibitors in metabolic diseases. As mentioned earlier, miR-574-3p and miR-9-5p have been demonstrated to be able to attenuate PRMT1 expression. These miRNAs may also prove to be suitable drug candidates in diseases associated with PRMT1 dysregulation. However, knowledge about the upstream and downstream mechanisms of the miRNA/PRMT1 interaction is still lacking. Therefore, in future research it is essential to conduct more detailed mechanistic studies on the miRNA/PRMT1 pathways.

In conclusion, PRMT1 makes for a truly appealing therapeutic target as it impacts a wide variety of cellular mechanisms and diseases. It is anticipated that therapeutic inhibition of its function may potentially benefit a lot of different patients in the future.

## Figures and Tables

**Table 1:** List of PRMT1-specific and -nonspecific inhibitors. ND, no data in the literature.

Name	Target(s)	Mechanism of Action	IC50 for PRMT1	Notes	Reference
AMI-1	PRMT1, -3, -4, -6	Competes with substrate	8.81 µM		[117]
Furamidine	PRMT1	Competes with substrate	9.4 μM		[118]
DCLX069	PRMT1	Interacts with substrate-binding pocket	17.9 µM		[119]
DCLX078	PRMT1	Interacts with substrate-binding pocket	26.2 µM		[119]
MS023	PRMT1, -3, -4, -6, -8	Competes with substrate	30 nM		[120]
PT1001B	PRMT1, -3, -4, -6, -8	Uncompetitive inhibitor for SAM	5.3 nM	Formerly known as compound 28d	[121,131]
GSK3368715	PRMT1, -3, -4, -6, -8	Competes with substrate	33.1 nM		[122]
Decamidine	PRMT1	Interacts with substrate-binding pocket	13 μM		[123]
TC-E-5003	PRMT1	ND	1.5 µM		[126]
C7280948	PRMT1	Interacts with substrate-binding pocket	12.8 µM		[127]
compound 9a	PRMT1	Uncompetitive inhibitor for SAM	3.5 μM		[128]
WCJ-394	PRMT1, -4, -8	Interacts with substrate-binding pocket	1.21 μM		[129]
Peptoid-based inhibitor P2	PRMT1	ND	8.73 μM		[130]

## Data Availability

No new data were created or analyzed in this study.
